# Immunological biomarkers of aging

**DOI:** 10.1093/jimmun/vkae036

**Published:** 2025-05-30

**Authors:** Fei Wu, Wei-Chieh Mu, Nikola T Markov, Matias Fuentealba, Heather Halaweh, Fiona Senchyna, Max N Manwaring-Mueller, Daniel A Winer, David Furman

**Affiliations:** Buck AI Platform, Buck Institute for Research on Aging, Novato, CA, United States; Buck AI Platform, Buck Institute for Research on Aging, Novato, CA, United States; Buck AI Platform, Buck Institute for Research on Aging, Novato, CA, United States; Buck AI Platform, Buck Institute for Research on Aging, Novato, CA, United States; Buck AI Platform, Buck Institute for Research on Aging, Novato, CA, United States; Buck AI Platform, Buck Institute for Research on Aging, Novato, CA, United States; Buck AI Platform, Buck Institute for Research on Aging, Novato, CA, United States; Diabetes Research Group, Division of Cellular and Molecular Biology, Toronto General Hospital Research Institute, University Health Network, Toronto, ON, Canada; Department of Laboratory Medicine and Pathobiology, University of Toronto, ON, Canada; Buck Institute for Research on Aging, Novato, CA, United States; Department of Immunology, University of Toronto, Toronto, ON, Canada; Buck AI Platform, Buck Institute for Research on Aging, Novato, CA, United States; Stanford 1000 Immunomes Project, Stanford University School of Medicine, Stanford, CA, United States

**Keywords:** biomarkers, immune aging, immunosenescence, inflammaging, human aging

## Abstract

The immune system has long been recognized for its critical role in the elimination of pathogens and the development of autoimmune diseases, but recent evidence demonstrates that it also contributes to noncommunicable diseases associated with biological aging processes, such as cancer, cardiovascular disease, neurodegeneration, and frailty. This review examines immunological biomarkers of aging, focusing on how the immune system evolves with age and its impact on health and disease. It discusses the historical development of immunological assessments, technological advancements, and the creation of novel biomarkers and models to study immune aging. We also explore the clinical implications of immune aging, such as increased susceptibility to infectious diseases, poor vaccine responses, and a higher incidence of noncommunicable diseases. In summary, we provide a comprehensive overview of current research, highlight the clinical relevance of immune aging, and identify gaps in knowledge that require further investigation.

## Introduction

Identifying biomarkers of aging is essential for understanding their effects on health, disease, and responses to longevity-promoting interventions.^1^ Immunological biomarkers can help differentiate between changes that are driven by aging and those specific to diseases, which is particularly important for conditions that predominantly affect the elderly. Immunosenescence, the gradual decline of the immune system associated with aging, impacts both innate and adaptive immune responses, leading to increased susceptibility to infections and a reduced response to vaccinations.^2^ In addition, inflammaging, a chronic low-grade inflammation associated with immunosenescence, increases the risk for nearly all noncommunicable diseases.^3^ Understanding the complex interplay between aging, immune function, and various external factors is essential for developing effective interventions.^3^

Novel biomarkers, such as the inflammatory aging clock (iAge), leverage deep learning to quantify chronic systemic inflammation and have shown strong correlations with multimorbidity and frailty.^4^ Additionally, immune cell proportion changes have been identified as significant biomarkers of aging.^5–8^ Recent studies using single-cell RNA sequencing (scRNA-seq) have revealed novel shifts in immune cell compositions, highlighting the complex and dynamic nature of immune aging. Moreover, lifestyle modifications, including diet and exercise, have shown promise in improving immune aging by positively impacting immunosenescence biomarkers.^9^ Additionally, certain drug interventions, such as metformin or mTOR inhibitors, offer targeted therapeutic benefits for conditions associated with immune aging.^10^^,^^11^

**Figure 1.**
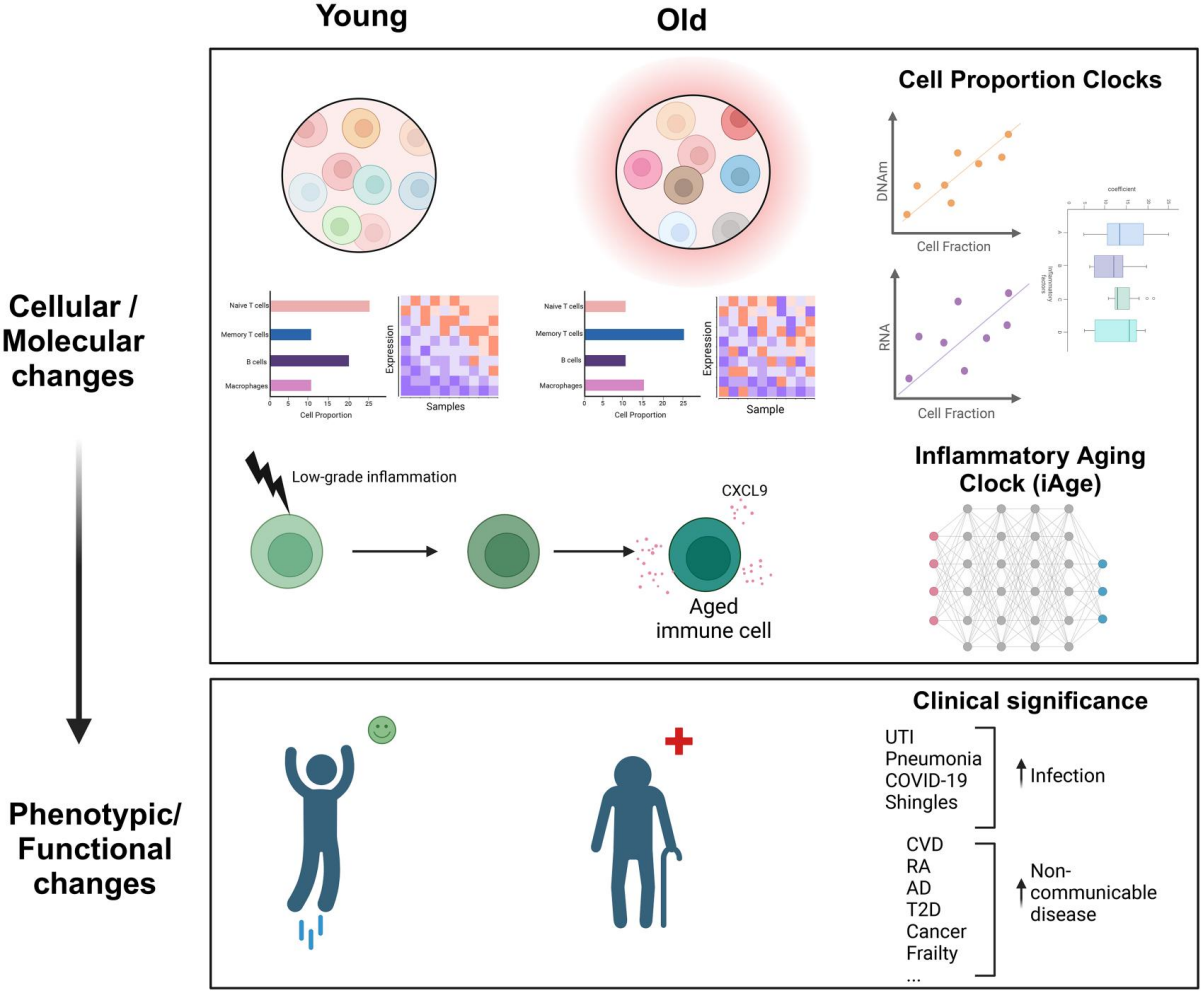
A schematic representation of novel immunological biomarkers of aging. This figure illustrates advanced techniques for modeling immune aging in humans, highlighting the cellular and molecular changes in the immune system that occur with aging. It also demonstrates the phenotypic and functional consequences of these changes, such as increased susceptibility to infections and noncommunicable diseases. AD, Alzheimer’s disease; CVD, cardiovascular disease; DNAm, DNA methylation; RA, rheumatoid arthritis; T2D, type 2 diabetes; UTI, urinary tract infection.

This review aims to update the understanding of the clinical significance of immune aging, including its phenotypic and functional changes, and model immune aging in humans (Fig. 1). We explore novel biomarkers and their roles, as well as potential strategies for mitigating the adverse effects of immunosenescence through targeted therapies and lifestyle modifications. By addressing these challenges and leveraging emerging technologies, such as the use of tonsil-derived organoid systems, we can improve the management of immune aging and enhance the healthspan of the aging population overall.

## Clinical significance of immune aging

### Higher prevalence of infectious disease in elderly

A significant disproportion in the prevalence of infectious diseases has been reported in older people compared with younger counterparts. One example includes urinary tract infections, which are also less likely to present with localized genitourinary symptoms in older adults.^12^ For respiratory diseases, at the peak of the COVID-19 pandemic, over 80% of deaths were in persons ≥60 y of age.^13^ Indeed, largely due to immune aging, older populations are disproportionately affected by many other infectious diseases in both incidence and severity.^14–16^ In SARS-CoV-2 infection, elevated levels of inflammaging-associated proinflammatory cytokines, such as interleukin (IL)-6, have been observed in the elderly, particularly older men, and these were associated with worse outcomes.^17^^,^^18^ Monocyte populations in the elderly have been linked to active *Mycobacterium tuberculosis* infections,^19^ and *Streptococcus pneumoniae*–induced antibody and phagocytic functioning has been shown to be reduced in older adults compared with the young.^20^ Additionally, aging of several components of the immune system has been linked to susceptibility to West Nile virus.^21^ In mice, human metapneumovirus infection was more severe in older mice that exhibited higher expression of programmed cell death protein 1 (PD-1) and other inhibitory receptors,^22^ and older mice were more susceptible to mousepox due to a decrease in total and mature natural killer (NK) cells.^23^ Finally, sepsis, a life-threatening multiorgan uncontrolled immune response to infection, is more likely to occur in older individuals, partly as a result of immunosenescence and inflammaging, and is often associated with reduced adaptive immune system functioning and overreactive innate immune cells.^4^^,^^24^

### Poor responses to vaccination

As a result of immunosenescence, older adults often exhibit diminished responses to vaccines, characterized by lower seroconversion rates and reduced antibody titers compared with younger individuals.^25^ Studies have shown that the repertoire of T cells and B cells becomes limited with age, reducing the body's ability to recognize and respond to new antigens. For instance, a study on the influenza vaccine revealed that elderly individuals had significantly lower hemagglutination inhibition titers postvaccination compared with younger adults, indicating a weaker immune response.^26^^,^^27^ Additionally, inflammaging can interfere with effective vaccine responses by disrupting the balance of pro- and anti-inflammatory signals necessary for a coordinated immune response. This compromised vaccine responsiveness poses a considerable public health challenge, as it leaves the elderly population more susceptible to infectious diseases. Strategies to enhance vaccine efficacy in older adults, such as the use of adjuvants, higher antigen doses, and novel vaccine formulations, are crucial to improving their immune protection and overall health outcomes.^28–30^

### Higher incidence of autoimmune disease

The pathogenesis of autoimmune disease is often multifactorial, comprising an interplay between factors such as genetics, lifestyle, external environment, and infection.^31^ The role of an aged immune system is also significant, and may be accelerated by the aforementioned factors.^32^ As the immune system ages, it can become self-reactive due to a shift to a proinflammatory environment and production of autoantibodies.^33^ Development of rheumatoid arthritis, a disease of chronic inflammation surrounding the joints, is linked to reduced and dysfunctional IgM-producing B cells, T cell metabolic and mitochondrial abnormalities, reduced T regulatory cells, and impaired expression and function of the immunosuppressive Foxp3 protein.^34–36^ Several immunosenescence markers have been associated with multiple sclerosis, an autoimmune disorder affecting the central nervous system, such as telomere shortening and shifts in T cell and NK cell populations.^37^^,^^38^ Furthermore, phenotypically distinct age-associated B cells, including CD21^low^ and IgD^−^CD27^−^ (double negative) B cells, have been implicated in several autoimmune diseases, such as systemic lupus erythematosus,^39^ rheumatoid arthritis,^40^ and multiple sclerosis.^41^ Decelerating immune aging has become increasingly important as the frequency of autoimmune disease rises globally, and the breadth of autoimmunity is yet to be fully elucidated.^42^

### Decreased immune surveillance and increased incidence of cancer

The aging immune system has a compromised ability to detect and eliminate malignant cells, a process known as immune surveillance, significantly contributing to the increased incidence of cancer observed in the elderly.^43^^,^^44^ This decline is thought to be primarily due to immunosenescence, which affects both the number and functionality of key immune cells such as NK cells and cytotoxic T lymphocytes (CTLs).^45–47^ Specifically, there is a reduction in the production of perforin and granzymes in CTLs and NK cells, which are essential for inducing apoptosis in tumor cells. Additionally, aging is associated with decreased expression of activating receptors such as NKG2D on NK cells, further impairing their cytotoxic function. Moreover, inflammaging creates a tumor-promoting environment by altering the tissue microenvironment through increased levels of cytokines like IL-6, tumor necrosis factor α (TNF-α), transforming growth factor β (TGFβ), and IL-1β.^48^ Some of these cytokines not only promote cancer cell proliferation, but also may suppress the function of immune cells. Another critical factor is the increased expression of PD-L1 on tumor cells and immune cells in the aged population, which binds to PD-1 receptors on T cells, leading to T cell exhaustion and diminished antitumor activity. This interaction is a major target of immunotherapies, but the efficacy of PD-1/PD-L1 inhibitors can be reduced in the elderly due to the overall decline in immune function.^49^ Remarkably, multiple studies have reported reduced aggressiveness of cancers in elderly adults compared with younger individuals due to slower tumor growth and metastasis.^50^ Understanding the mechanisms behind decreased immune surveillance in the elderly is essential for developing strategies to enhance anticancer immunity, such as boosting NK cell and CTLs function, modulating the inflammatory and mechanical environment to reduce tumorigenesis and improving cytokine and chemokine signaling pathways.^51^ These strategies could significantly improve cancer prevention and treatment outcomes in this vulnerable population, ultimately contributing to better overall health and longevity.

### Rise in inflammaging increases risk for nearly all noncommunicable disease

Age-associated dysregulation of the immune system not only impairs the integrity of host defense system, but also contributes to functional decline of organs and increasing susceptibility to and progression of diseases during aging.^52^^,^^53^ Notably, both the systemic and local environments within organs, including the brain,^54^ heart,^55^ adipose tissue,^56^ lung,^57^ liver,^58^ and kidney,^59^ shift toward a proinflammatory state during normal aging, posing a much greater risk of noncommunicable diseases.

In the aging brain, neurons and glial cells become senescent and secrete elevated levels of proinflammatory factors that drive neuroinflammation and functional decline.^60–63^ These proinflammatory cytokines disrupt the production of plasticity-related molecules such as BDNF (brain-derived neurotrophic factor) and IGF-1 (insulin-like growth factor 1), which impairs synaptic plasticity and neuronal function, causing brain damage and leading to neurodegenerative diseases like Alzheimer’s disease, Parkinson’s disease, and stroke.^64^^,^^65^ Similarly, in the aging heart, hypoxic and hypertrophic cardiomyocytes produce proinflammatory factors, and the slow repair of damaged cardiomyocytes leads to cardiac fibrosis and ultimately heart failure.^66^ Proinflammatory factors, such as MCP-1 (monocyte chemoattractant protein-1) and TNF-α, aggravate atherosclerosis by recruiting monocytes and converting them into lipid-containing, foamy macrophages.^67^ These activated foamy macrophages secrete senescence-associated secretory phenotype (SASP) and create a feedforward loop that promotes plaque instability and exacerbates atherosclerotic progression.^67^

Systemic chronic, low-grade inflammation also interferes with insulin signaling and drives insulin resistance and its associated lipotoxicity, further enhancing inflammation and tissue degeneration.^68^^,^^69^ This vicious cycle contributes to the pathogenesis of type 2 diabetes and other inflammaging-related diseases such as cardiovascular disease.^68^ Additionally, inflammaging induces structural damage and extensive tissue fibrosis in the liver, kidneys, and lungs, which diminishes organ function and facilitates the onset of chronic conditions such as chronic liver disease, chronic kidney disease, and chronic obstructive pulmonary disease.^52^ Ultimately, the coexistence of diminished adaptive immunity and chronic, low-grade inflammation leads to the onset of age-associated, noncommunicable diseases and higher risk of morbidity and mortality in the elderly compared with the young.^3^

## Phenotypic and functional changes observed with aging

### Historical perspective

Although blood phenotyping can be traced back to the invention of the hemocytometer by Karl Vierordt in 1852 and the Romanowsky stain that allowed the differential count of blood cell types, these techniques were complicated and time consuming. It is with the advent of the Coulter counter in 1953 that the complete blood count (CBC) test entered the clinical domain and larger studies revealed its usefulness as a biomarker of aging.^70^ The main parameters of the CBC are the numbers of white blood cells, red blood cells, platelets, and hematocrit and hemoglobin. These parameters allow evaluation of the progression of diseases of the bone marrow and blood, such as leukemia, anemia, thrombocytosis, polycythemia, etc. Several large cohort studies have provided reference levels for phenotypic characterization^71–74^ with the National Health and Nutrition Examination Survey constituting the major effort in the field. Since then, multiple scores have been developed that connect parameters of the CBC with aging and risk of disease.^75–78^ Generating these large datasets of age referenced normal values for an important clinical parameter like the CBC enables better diagnosis and treatment outcome prognosis.^79^ Interestingly parameters associated with the CBC have been reported to have seasonal variation patterns that are to be taken into account if these parameters are used in aging studies.^80^

### Specific immune cell–type changes during aging

The complexity of the immune system is perplexing, as it contains nearly 20 times more cells than the brain (1.8 trillion) across the whole body and weighs 1.2 kg (2.6 pounds). It forms an intricate network of multiple cell types controlling the complex response to novel or recognized infections and defense against internal abnormalities such as cancer cells.^81^ Much of the capacity of the immune system to perform these functions resides in the subtle control of the proportions and density of immune cells with different phenotypes forming the landscape of human immune cells.^82^ Two major technologies have allowed for the analysis and exploration of the distribution of immune cell phenotypes. The first method, flow cytometry, consists of the labeling of different cell surface markers with fluorescent antibodies followed by a cell-by-cell evaluation of the fluorescent signal intensity for each marker. The gating procedure allows the quantification of the incidence of up to 40 different phenotypes. An alternative technology, cytometry by time of flight, uses the multiplexing capabilities of heavy metal isotopes to detect the different phenotypes. It is capable of differentiating up to about 70 different phenotypes. The main shortcoming of this technique is that analysis requires destruction of the cell sample. Several studies have reviewed the immune landscapes generated via either method and found them quite comparable.^83–85^ This has allowed the establishment of normative ranges for the healthy human immune system.^86^^,^^87^ The major cell types of the immune system are lymphocytes (∼40% of immune cells), neutrophils (∼40% of immune cells), macrophages (∼10% of immune cells), eosinophils (∼2%–5% of immune cells), basophils (∼0.5%–1% of immune cells), and dendritic cells (∼1%–2% of immune cells). The roles that these cells play in the immune response are split into innate and acquired immunity. Both of these systems have protracted development and decline throughout the individual lifespan.^88^

The cellular profiles of the innate immune system of younger (<50 y of age) and older (>50 y of age) individuals express several remarkable differences. There is a significant decrease in the population of classical monocytes, double negative T cells, T lymphocytes, HLA-DR–negative T cells, and NK T cells, while in parallel, other cell populations increase in incidence (NK cells, all monocytes, HLA-DR–positive T cells, and intermediate monocytes).^89^ Out of the innate changes, special attention is attributed to the aging related changes in the different subpopulations of NK cells. These cells have multiple roles involving cytotoxicity toward transformed cells, either cancerous or virus infected.^90^ Their role in the production of inflammatory cytokines and chemokines is reviewed further subsequently. In normal conditions, the innate immune system provides a first line of response after immediate exposure to pathogens. During aging, the return to a quiescent state is altered, and a state of lingering chronic inflammation is present and accompanies altered cell-mediated responses. This is believed to play a role in making older adults more susceptible to infection.^91^ On the other hand, it has been shown that the CD57^+^ population of CD56dimCD16 bright NK cells represents a population of cells with low proliferative ability and lower response to stimulation with IL-2 but does not constitute a population of “anergic exhausted” cells. They represent a population of long-lived mature cells that have encountered pathogens and represent “memory” NK cells.^92^ These patterns of immune aging are differentiated in males and females after the age of 65 y. Men express higher innate and proinflammatory activity and lower adaptive activity.^93^

One of the expressions of the aging immune system is observed in population level analyses. While in younger participants the T-to-B-cell ratio is quite stable, within a range of 9.2 to 12.4, in older participants this ratio is much more heterogeneous (6.4–26.1).^89^ Lymphocytes aging contains lots of similarities between CD8^+^ and CD4^+^ cells, with the progression of naïve to central memory and effector memory to the end-stage nonproliferative effector cells.^94^^,^^95^ Analysis of T cell subsets shows the drastic reduction in the naïve phenotype and expansion of the memory cell phenotype; this change is explained by the continued thymus involution since puberty, compensatory homeostatic proliferation of T cells, and repeated exposure to different antigens.^96^^,^^97^ The complexity of immune aging is further exacerbated by the fact that (1) the cell proportions change and (2) there are alterations in the expression levels of a variety of metabolism genes and in the surface markers defining the different immune cells.^98^^,^^99^

### Canonical cell functions that change with age

Aging not only significantly impacts the composition and molecular profiles of immune cells, but also profoundly affects their functionality.^53^ One hallmark of the aging immune system is the dysfunction of hematopoietic stem and progenitor cells (HSPCs). Aged HSPCs experience oxidative stress, become senescent, and exhibit significant functional changes. These changes include impaired self-renewal capacity with an expansion in number, decreased cell polarity, and a differentiation bias skewed toward myeloid and megakaryocytic lineages.^100–102^ The proinflammatory bone marrow microenvironment, characterized by elevated levels of cytokines and growth factors such as IL-1 and TNF-α, exacerbates the imbalance of myelopoiesis and lymphopoiesis during aging.^103^^,^^104^ Moreover, age-associated clonal expansion of HSPCs, caused by mutations in epigenetic regulators such as *DNMT3A*, *TET2*, and *ASXL1*, drives inflammatory responses in macrophages and mast cells.^105^ This leads to subsequent immune dysfunction, increasing the risks of malignancies, coronary heart disease, and cardiovascular-related mortality.^105^ Altogether, these age-related changes in HSPCs appear to form the basis of immunosenescence and inflammaging in the aging immune system.

#### Innate immune cells

Aged HSPCs give rise to functionally compromised immune cells that exacerbate immune system aging and organ dysfunction.^100^ Myeloid cells, including macrophages, granulocytes, and dendritic cells (DCs), undergo functional changes with age, although to a lesser extent than T and B cells.^106^

Monocytes and macrophages are central to the development of inflammaging and immunosenescence in the aging immune system. In aged individuals, monocytes and macrophages lose their phagocytic capacity to remove pathogens, senescent cells, tumor cells, and other tissue damage, leading to unresolved and sustained inflammation.^107–109^ Additionally, age-related mitochondrial dysfunction and a decline in mitophagy in macrophages further promote the activation of the NLRP3 inflammasome and STING (stimulator of interferon genes) signaling, leading to the release of proinflammatory factors in mice.^110^^,^^111^ Consequently, aged macrophages produce more proinflammatory cytokines, such as TNF-α, IL-6, and IL-1β, while reducing the production of the anti-inflammatory cytokine IL-10.^109^^,^^112^ This imbalance in cytokines contributes significantly to the chronic, low-grade inflammation observed in aging.

Aged neutrophils exhibit functional declines, including abnormal formation of neutrophil extracellular traps, reduced peroxide production, and diminished activities in phagocytosis and killing of bacteria.^113^ Dysregulated trafficking resulting from downregulated CXCR2 (the receptor of CXCL1), together with tissue infiltration of neutrophils, leads to augmented tissue inflammation, senescence, and injuries in old mice.^114^ Aged DCs contribute to inflammaging by producing more proinflammatory cytokines and lower levels of anti-inflammatory cytokines like IL-10.^106^ Impaired phagocytosis and migration of aged DCs, along with the decreased T cell crosstalk with plasmacytoid DCs, results in diminished vaccine response and increased susceptibility to infections in the elderly.^115^ In addition, aged DCs exhibit increased reactivity to self-antigens and a loss of tolerance, which can lead to autoimmunity and inflammation.^116^ In NK cells, aging deteriorates cytokine production and cytotoxic activity through the downregulation of cytotoxicity-activating receptors and perforin secretion.^117^^,^^118^ The reduced perforin may impair senescent cell clearance, fueling more senescent cell cytokine production and a positive feedback loop of chronic inflammation. The aged host environment is responsible for the defective priming and maturation of NK cells in mice.^119^ These dysfunctions in NK cells lead to impaired antiviral and antitumor immunity, as well as to reduced vaccination efficacy in older adults. Taken together, these age-related functional declines in innate immune cells lead to increased inflammation, impaired immune responses and a higher risk of infections, autoimmunity, and organ dysfunction in the elderly.

#### Adaptive immune cells

Thymic involution leads to decreased production and functionality of naïve T cells, with a much greater impact on CD8^+^ naïve T cells than CD4^+^ naïve T cells, in part due to homeostatic proliferation that preserves the CD4^+^ naïve T cell compartment.^50^^,^^52^^,^^120^ Aging results in a loss of CD8^+^ naïve T cells and an increase in the memory subset, characterized by remodeling and clonal expansion.^121^^,^^122^ Age-associated CD8^+^ T cells have been recently discovered in both mice and humans.^121^ These age-associated CD8^+^ T cells are found in multiple tissues in old mice, expressing high levels of exhaustion markers TOX and PD-1 and producing proinflammatory factors such as granzyme K (GZMK) and CCL5.^121^ GZMK secreted by age-associated CD8^+^ T cells further enhances the release of SASP from senescent cells.^121^ In humans, the age-associated GZMK^+^ CD8^+^ effector memory T cells share transcriptional and epigenetic signatures with their mouse counterparts, suggesting their role in driving inflammaging.^121^

Aged naïve CD4^+^ T cells develop functional defects, including reduced proliferation and responsiveness to antigen activation, and suppressed production of IL-2.^123^^,^^124^ Inappropriate activation of aged CD4^+^ T cells leads to aberrant and incomplete differentiation of T helper 1 and 2 effector cells, resulting in an imbalanced subset of different T helper cells in mice with a skew toward T helper 17 cells, driving inflammaging and autoimmunity.^125^^,^^126^ Regulatory T cells, a subset of T helper cells that are immunosuppressive and responsible for self-tolerance, increase with age and shift toward glycolytic metabolism, leading to cellular senescence and potentially increased infections and neoplastic malignancies in older adults.^127–129^ Another recently identified subset of age-related CD4^+^ T cells are cytotoxic CD4^+^ T cells, characterized by the expression of *Eomes* and *Gzmk*, and the release of proinflammatory cytokines (interferon γ [IFN-γ] and TNF-α) and cytotoxic factors (GZMB and perforin).^130^^,^^131^ Notably, cytotoxic CD4^+^ T cells are more abundant in supercentenarians via clonal expansion, suggesting an immune adaptation in late aging.^131^ Moreover, cytotoxic CD4^+^ T cells are also more prevalent in moderate COVID-19 cases, indicating their protective role against viral infections.^132^

T cell senescence, marked by the downregulation of costimulatory molecule CD28 and consequent hyporesponsiveness to T cell reporter (TCR) signal transduction, is another contributor to immunosenescence and inflammaging.^52^^,^^133^ Senescent-like CD28^−^ T cells, especially CD8^+^ T cells, increase with age and exhibit immunosuppressive properties that disrupt the antigen-presenting function of DC, antigen-specific T cell responses, and T cell cytotoxicity. The expansion of CD8^+^CD28^−^ T cells is considered a risk factor for chronic viral infections, autoimmune diseases, and cancer.^52^^,^^133^ Additionally, the decline in TCR diversity, caused by reduced naïve T cell output and clonal expansion of memory T cells, is directly linked to immunosenescence.^50^^,^^52^^,^^134^ Single-cell RNA sequencing of paired α-chains and β-chains has confirmed age-associated TCR repertoire attrition in mice and humans.^121^ This age-associated decline in T cell repertoire impairs their responsiveness to neoantigens and increases susceptibility to novel infections of the elderly.

Age-related changes in B cells weaken the antibody response to vaccination and infection in the elderly population.^52^ B cell receptor (BCR) sequencing reveals reduced BCR diversity and enhanced clonality in older individuals.^135^^,^^136^ The reduced capacity for class-switch recombination, somatic hypermutation, and affinity maturation in aged B cells leads to diminished antibody titers, affinity, and neutralization capacity against pathogens and in response to vaccines in the elderly.^137–139^ Additionally, aged B cells show increased autoantibody production in part due to reduced expression of autoimmune regulator and autoimmune regulator–dependent self-antigen genes in thymic B cells.^140^ These age-related defects in B cells lead to weakened vaccination and infection responses and a higher risk of autoimmunity in the elderly. Age-associated B cells, a unique subset that emerges during aging, are characterized by memory markers and late-stage exhaustion.^141^^,^^142^ Functionally, these cells promote inflammaging and inhibit B lymphopoiesis by secreting proinflammatory mediators, such as IFN-γ and of TNF-α,^143^ and contribute the onset of autoimmunity through IgG autoantibody production.^144^ The accumulation of dysfunctional B cells is a key driver of the deterioration of aging immune system, contributing to immunosenescence, inflammaging, and autoimmunity.^96^

Given the profound impact of aging on immune cell composition and function, it is crucial to employ advanced techniques to thoroughly understand these changes. Phospho-flow cytometry is a powerful tool for studying age-related changes in immune system dynamics, enabling detailed and unbiased profiling of immune function through assessing phosphorylation states of intracellular signaling proteins.^145^ It offers a comprehensive view of cellular signaling pathways and their dynamic changes in response to stimuli.^146–148^ For example, intracellular levels of phosphorylated STAT proteins serve as a functional readout of cytokine stimulation and inflammation.^149^^,^^150^ Phosphorylation of IRF7 and TBK-1, critical antiviral signaling pathways responsible for type I IFN production, is blunted in DCs and monocytes upon stimulation in aged individuals.^151^ This, along with diminished IFN-α secretion and phagocytosis, suggests an impaired innate immune function that might interfere with vaccination efficacy and defense against infections.^151^ These findings underscore the versatility of phospho-flow cytometry in identifying changes in intracellular protein phosphorylation, potentially serving as biomarkers for monitoring dysregulated immune responses in aging.

### Novel biomarkers of immune aging

#### Development of metabolomics for evaluation of age-related changes in immune cell metabolism

Metabolomics identifies key biomarkers by analyzing shifts in metabolites as the terminal products of the genome, offering a close connection to cellular and organismal phenotype and functional status.^152^^,^^153^ The untargeted approach, through the unbiased measurement of thousands of metabolites, particularly facilitates a comprehensive understanding of the metabolic networks affected by infections or diseases.^154^ Indeed, various metabolic alterations due to infections have been established using untargeted metabolomics. These include abnormal nucleotide metabolism in older adults with high levels of inflammasome genes,^155^ glycerophospholipid metabolism and glutamine and glutamate metabolism in HIV infection,^156^ and tricarboxylic acid cycle and arginine metabolism in patients with varying severity of COVID-19.^157^

Metabolomics studies commonly utilize liquid samples, such as plasma and serum, due to their accessibility, to infer metabolic changes associated with aging or diseases.^158^ Recently, a method called SCENITH (Single-Cell Energetic Metabolism by Profiling Translation Inhibition) has been developed to study energetic metabolism at the single-cell level via flow cytometry.^159^ This technique allows for the detailed analysis of cellular metabolic activities orchestrated by multiple cell types, providing unprecedented insights into the metabolic states of individual cells within heterogeneous populations, such as human meningioma tumors.^159^ Future studies using SCENITH could significantly advance our understanding of immune aging by enabling the detailed profiling of metabolic changes in different immune compartments.

#### Inflammatory aging clock

Having explored the changes in cell composition and canonical cell functions with age, it is crucial to delve into the identification of novel biomarkers that can provide deeper insights into the mechanisms of immune aging. Recent advancements highlight the inflammatory aging clock (iAge)^4^ as a novel biomarker for immune aging, leveraging deep learning to quantify systemic chronic inflammation (Fig. 1). Developed from blood immune biomarker analysis of over 1,000 individuals, iAge correlates with multimorbidity, frailty, and cardiovascular aging. The chemokine CXCL9 emerged as a significant contributor, linked to poor vascular function and endothelial cell senescence. Silencing CXCL9 in aged endothelial cells reversed several aging phenotypes, underscoring its potential as a therapeutic target. The iAge metric offers a promising tool for predicting age-related health outcomes and informing strategies to enhance healthy aging.

#### Cell proportion clock

In addition to the inflammatory factors, recent studies have underscored immune cell proportion changes as significant biomarkers of aging. Leveraging longitudinal follow-up on a large human cohort, Alpert et al.^160^ identified that a linear model represents the change in characteristic cell proportions during aging. A major characteristic difference between the young and aging population is the relative stability of the immune cell proportions in the young cohort, while the older individuals have branching departures from the youthful norm that represent different ageotypes. The characteristic pattern of cell proportions is an increase in CD8^+^ effector memory T cells and mature NK cells CD56^+^CD16^+^CD3^−^ and a decrease in a number of cell types including naïve CD8^+^ T cells, mature B cells, naïve CD4^+^ T cells, central memory CD4^+^ T cells, eosinophils, basophils, effector memory CD4^+^ T cells, and central memory CD8^+^ T cells. The changing landscapes of immune cell proportions with age mean that epigenetic analyses of peripheral blood mononuclear cells (PBMCs) and subsequently developed clocks would be affected by these cell identities. For example, comparing the human naïve CD8^+^ T cells with the effector memory CD8^+^ T cells that have anticorrelated trajectories shows that the former are 15 to 20 y younger in epigenetic age than the latter. To overcome this issue, the recently reported IntrinClock does not change with the different proportions of cell types, and it can be driven in the forward direction with models of replicative senescence or reversed via OSKM-mediated reprogramming.^161^

Moreover, Zhu et al.^6^ developed an scRNA-seq–based aging clock using PBMCs, identifying shifts in naive CD8^+^ T cells and memory CD4^+^ T cells as key indicators of biological age. This model revealed unique immune cell compositions in supercentenarians, correlating with their longevity. Similarly, Zhang et al.^7^ demonstrated that changes in naive and memory lymphocyte subsets can predict epigenetic age acceleration. Another study integrated machine learning with scRNA-seq data to create aging clocks that account for immune cell heterogeneity and capture both aging-accelerated and aging-delayed features by analyzing the dynamic changes in cell proportion and functions.^8^ These models collectively underscore the potential of combining cellular and molecular biomarkers to assess immune aging accurately.

In summary, the identification and analysis of novel biomarkers such as changes in metabolomics, iAge, immune cell proportion changes, and gene expression patterns provide valuable insights into the mechanisms of immune aging. These biomarkers not only offer predictive insights into age-related health outcomes, but also serve as valuable indicators for identifying therapeutic targets to address immunosenescence. Moreover, these efforts align closely with the mission of the Biomarkers of Aging Consortium, which aims to identify and validate reliable biomarkers that reflect biological aging.^1^ Through a combination of cellular, molecular, and metabolic biomarkers, the Consortium's work aims to set new standards for assessing aging and accelerating the development of interventions to extend healthspan and improve aging-related clinical outcomes.

## Drivers of immunosenescence and inflammaging

We have described the different outcomes of immunosenescence and its hallmarks, though these are driven by multiple factors linked to aging. As a dynamic process that develops over decades, it is crucial to consider the interplay between organ aging and immunosenescence.^52^ Immune cells, being part of the circulatory system, are not confined to a single cellular niche but interact reciprocally with multiple body organs. Certain drivers of inflammaging are tissue specific, for example, adipose tissue significantly influences immune responsiveness through adipokine secretion. In lean individuals, adipokines and resident immune cells promote a balanced immune response that avoids hyperactivation, whereas obesity and aging alters the adipokine profile, shifting the immune system toward low-grade chronic inflammation, contributing to metabolic disorders and the aging process.^162^^,^^163^ In contrast, some inflammatory drivers like SASP and immune cell senescence are shared across multiple tissues. For instance, aging skin, a barrier organ housing various cell types including immune cells, accumulates senescent cells that produce SASP, which may drive immunosenescence in resident immune cells.^164^ In addition, aging and age-associated chronic diseases perturb the composition of the immune system within the intestines, which is associated with microbial dysbiosis, changing gut-derived metabolites, and potentially intestinal barrier disruptions, all fueling systemic chronic inflammation.^165^^,^^166^ Additionally, the involution of primary lymphoid organs, such as the thymus and bone marrow, directly impacts immune system aging. Concurrently, senescence of peripheral lymphoid organs like the spleen and lymph nodes leads to age-associated changes in immune cell phenotypes and cytokine signaling.^2^ Collectively, the aging of multiple organs and tissues, particularly hematopoietic and immune organs, disrupts immune homeostasis, contributing to immunosenescence and inflammaging.^167^

These high-level mechanisms can be tracked down to the subcellular processes driven by the primary hallmarks of aging^168^ with major contributions from mitochondrial impairment, telomere shortening, genetic instability, and altered proteostasis.^53^ Proper bioenergetic status is critical for maintaining an immune cell inflammatory status. Impaired mitochondrial function leads to elevated production of reactive oxygen species, stress signals, and impaired oxidative phosphorylation, which can sometimes lead to a favoring of inflammation-linked glycolytic metabolism.^169^ In particular, mitochondrial alterations in aged T cells maintain them in activated state, hindering the resolution of inflammation.^170^ Moreover, cellular senescence and metabolic dysfunction are tightly intertwined drivers of inflammaging, impacting energy-sensing signaling pathways such as sirtuins, AMPK, and mTOR, and compromising mitochondrial health. Tissue-resident macrophages and the NLRP3 inflammasome connect sterile and metabolic inflammation with cellular senescence and tissue dysfunction, emphasizing the role of metabolic regulation in immune cell aging.^171^ Interestingly, restoring mitochondrial function could be a future avenue for cellular rejuvenation.^172–174^

Telomere shortening and DNA damage are critical drivers of immunosenescence and inflammaging.^53^ Impaired DNA repair in the hematopoietic lineage, particularly due to the loss of *Ercc1*, has been linked to immunosenescence and accelerated immune aging in mice.^175^ This deficiency not only leads to senescence within the immune system, but also results in the degeneration of distant solid organs.^175^ Moreover, telomeres are particularly prone to DNA damage, which accelerates their shortening.^176^ Telomere dysfunction drives mitochondrial abnormalities, increases oxidative stress, and ultimately activates the NLRP3 inflammasome in murine macrophages.^110^ Telomere shortening in T cells induces senescence, impairs proliferation and function, and simultaneously increases the release of proinflammatory cytokines such as IL-6 and TNF-α.^122^^,^^177^ Strikingly, Lanna et al.^178^ demonstrated that telomere transfer from antigen-presenting cells to some CD4^+^ T cells (naïve and central memory) prevents T cell senescence and promotes long-lasting immune protection. Overall, telomere shortening and the accumulation of DNA damage throughout aging promote inflammation and weaken immune resilience.

A decline in autophagy and proteostasis is a pivotal contributor to immunosenescence and inflammaging, intricately linked to dysfunctional protein and organelle quality control. The reduction in autophagic flux impedes the clearance of defective mitochondria, thereby increasing mitochondrial DNA and reactive oxygen species release, both of which are potent inducers of the NLRP3 inflammasome.^179^ Additionally, reduced autophagy may slow the disposal of pattern recognition receptor ligands. Simultaneously, a decline in proteostasis machinery, including proteasomes and chaperones, leads to the accumulation of misfolded proteins that activate stress-related signaling pathways, such as the unfolded protein response and nuclear factor κB pathway, amplifying inflammatory cytokine production.^180^

Additionally, the exposome, the cumulative environmental exposures experienced over a lifetime, collectively contributes to the development of immunosenescence and inflammaging.^3^ The exposome contains a wide range of factors, including chronic infections, lifestyle habits, and environmental exposures, all of which contribute to immune dysregulation. Persistent infections, such as cytomegalovirus and hepatitis C, can disrupt immune homeostasis, leading to elevated inflammatory markers. Furthermore, modern lifestyle factors, particularly physical inactivity and poor dietary patterns, exacerbate inflammatory processes. Physical inactivity reduces the production of anti-inflammatory myokines released during muscle contraction, promoting insulin resistance and metabolic dysfunction, while diets rich in processed foods and low in fiber contribute to gut microbiota dysbiosis, triggering systemic inflammation.^181^ Social and cultural changes associated with industrialization, including increased psychological stress, disrupted circadian rhythms, and inadequate sleep, further heighten the risk of systemic chronic inflammation. Finally, the increasing exposure to environmental pollutants and industrial chemicals, such as phthalates and airborne toxins, significantly impacts molecular pathways that regulate inflammation.^3^

Altogether, these mechanisms culminate in the development of low-grade chronic inflammation in aging. A significant consequence of this chronic inflammation is the immune system’s diminished capacity to mount an acute response. This phenomenon is linked to defects in the JAK-STAT signaling pathway, which becomes dysregulated as cells are persistently exposed to proinflammatory mediators such as IL-6 and C-reactive protein.^145^ These chronic stimuli lead to elevated baseline levels of phosphorylated STAT proteins, which reduce the flexibility of immune cells to respond effectively to acute cytokine bursts, such as those seen during infection or injury. In older individuals, the immune system's chronic exposure to inflammatory cytokines prevents the proper resetting of this pathway, thus blunting the acute immune response and contributing to the immune system's overall decline in effectiveness during aging.^145^

## Modeling immune aging in humans to identify interventions

Building on the identification of novel biomarkers of immune aging and the understanding of the mechanisms behind it, the next critical step is to establish models that accurately reflect the cellular, structural, and functional aspects of the human immune system. While traditional inbred mouse models have been valuable, their limitations in predicting human responses are apparent.^182^^,^^183^ Though large-scale human studies offer insights, cross-sectional designs often lack the capacity to capture individual variability, and longitudinal studies prove resource-intensive. Therefore, improving and innovating new models and methodologies is essential for identifying effective interventions for immune aging.

**Figure 2.**
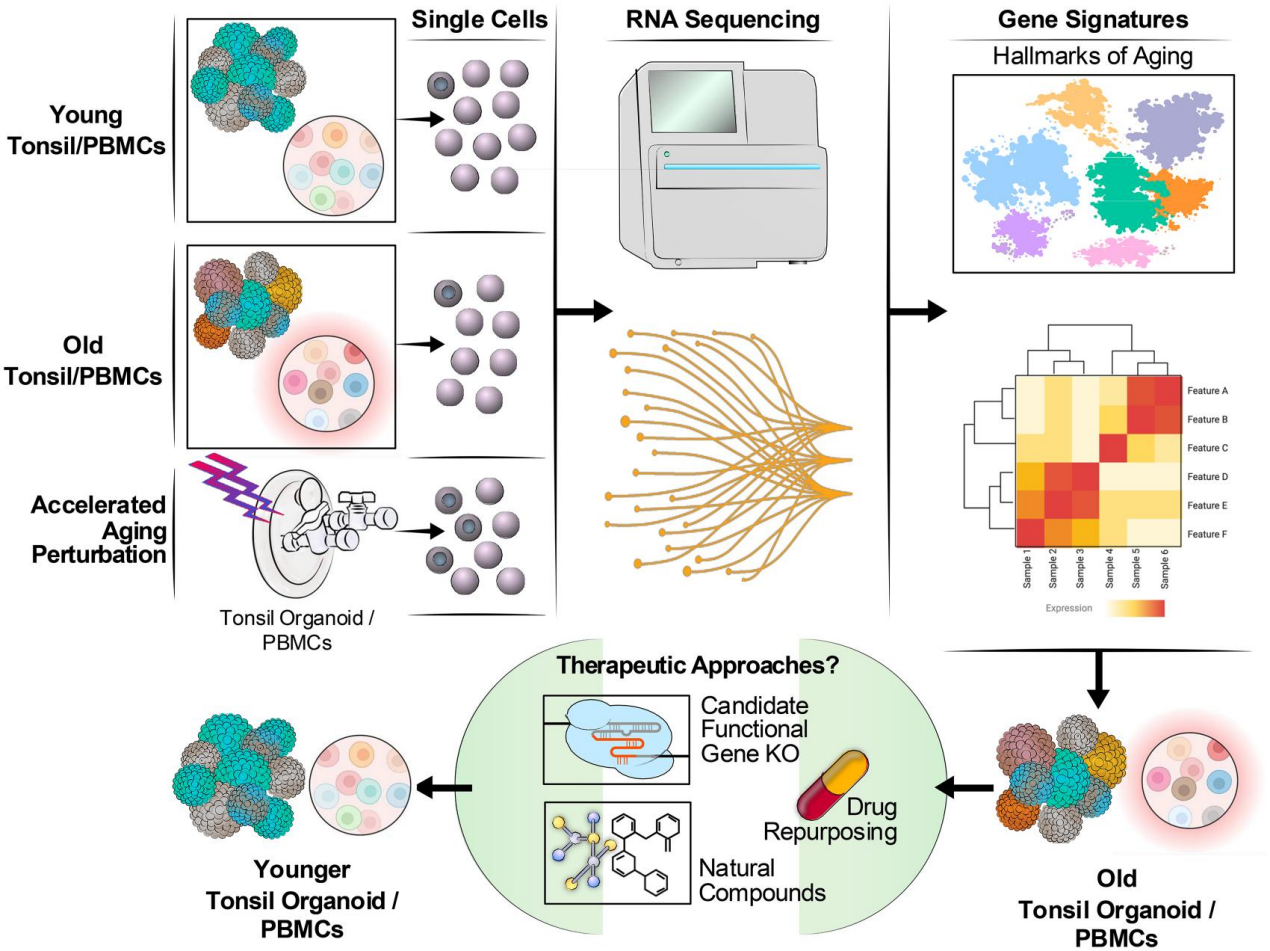
A platform for modeling immune aging in humans using human tonsil-derived organoids or PBMCs. This system employs accelerated aging perturbations, such as microgravity, to simulate immune aging. Subsequent omics analyses, including RNA sequencing, are conducted to identify potential interventions that can mitigate the detrimental effects of aging on the immune system. KO, knockout.

A promising alternative emerges with immune tissue organoids, such as tonsil-derived organoids, adeptly recapitulating key features of human germinal centers while preserving individual variability.^184^ This scalable, accessible, and genetically manipulable platform has great potential and may rapidly improve areas of translational research, such as vaccine development and immune aging (Fig. 2).

A key advantage of organoids is their ability to perform “clinical trials in a dish,” accounting for individual and population-level variability in immune responses. This is particularly relevant for improving vaccine approaches, especially for older adults who often exhibit varied responses.^185^ Additionally, organoids enable the study of age-related changes in immune cell populations and function, as demonstrated by research identifying phenotypic shifts in T cells from older individuals that can be reversed in the organoid environment.^186^

Furthermore, organoids offer a unique platform for studying the impact of different environmental factors, such as lifestyle changes or nutrition, as well as more challenging environments that may be linked to aging, like lower gravity on immune function. Interestingly, by culturing PBMCs or organoids in simulated microgravity, researchers can investigate the effects of altered mechanotransduction on immune cell signaling and communication, as was recently done in cell culture.^187^ Indeed, single-cell analysis of human PBMCs following 25 h of simulated microgravity could recapitulate a number of aging factors on the immune system, including basal monocyte inflammatory change, mitochondrial dysfunction, and reduced adaptive immune responses and IFN production to TLR7/8 agonist.^187^ The insights gained from these microgravity studies hold significant implications for both space biology and the understanding of aging processes on Earth.^188^

### Drug and lifestyle that improve immune aging

Immunosenescence is a universal aspect of human aging, yet clinical studies have demonstrated that both pharmacological and lifestyle interventions can mitigate its effects. For instance, Mannick et al.^11^^,^^189^ conducted 2 trials showing that everolimus (RAD001), an mTORC1-specific inhibitor, enhances vaccine effectiveness, elevates antibody levels, decreases infection rates, and reduces T cell exhaustion in the elderly. However, a third trial found no effect on the reduction of respiratory tract infections, regardless of vaccination status.^190^ Similarly, a recent 20-wk pilot trial in nondiabetic older adults revealed that metformin, commonly used to treat type 2 diabetes, reduces CD4^+^ T helper cell exhaustion in response to the influenza virus.^10^ Additionally, studies have shown that zinc supplementation, which is often low in older adults, can lower infection incidence in healthy individuals^191^ and improve vaccine response.^192^

Thymic atrophy, which accelerates immune aging by reducing naïve T cell production, can weaken the immune response. In this context, studies have supported the safety and effectiveness of IL-7 administration in expanding naive T cell populations.^193^ Physical activity also plays a crucial role in enhancing immune function in older adults, leading to improved migratory dynamics^194^ and phagocytosis^195^ of neutrophils, increased leukocyte numbers,^196^ and a higher proportion of central memory CD4^+^ and effector memory CD8^+^ T cells, as well as to longer telomere length.^197^ Furthermore, studies have demonstrated that exercise can enhance the expression of CD28, a crucial surface molecule in T cells, whose decline with age is a feature of immunosenescence and loss of age-related decline in intrinsic capacity.^99^^,^^198^

Regarding nutrition, probiotics are among the most validated dietary components with immunomodulatory properties. Six months of probiotic supplementation have been shown to decrease the percentage of CD8^+^CD28^null^ T cells, increase the CD4/CD8 ratio, and elevate the proportion of naive CD4 and CD8 cells.^199^ Additionally, probiotic consumption improves influenza vaccine response^200^ and reduces the risk of respiratory infections.^201^ Caloric restriction or intermittent fasting may also offer some benefits on immune functionality. Work has shown that a 14% caloric restriction for 2 y in healthy humans can improve thymopoiesis, and healthspan parameters in mice.^202^ Intermittent fasting may also change gut microbial composition, reduce inflammation, and improve glucose metabolism, all features that can improve healthspan.^96^

## Conclusions

The study of immunological biomarkers of aging has revealed important insights into the cellular and molecular changes that occur with age. Techniques such as flow cytometry and mass cytometry have been crucial in identifying age-related alterations in immune cell subsets and functions, providing a detailed understanding of immunosenescence. The development of cell proportion clocks, inflammatory clocks, and metabolomics approaches has further enhanced our ability to quantify biological age and assess the health of the immune system. These advancements offer promising opportunities for practical applications, such as improving the accuracy of biological age estimation and guiding personalized interventions to enhance immune function in the elderly.

Recent studies have indicated a loss of cell identity during aging.^203^ Cellular senescence has been implicated in this process, as it can act both as a barrier and a driver of cellular plasticity, influencing cell identity changes.^204^ However, in many immune cell types, direct evidence of cell identity alteration remains limited. Moreover, a decline in tissue overall stemness with aging is observed, as most tissues show a significant negative correlation between age and stemness scores.^205^ In contrast, hematopoietic stem cells (HSCs) derived from older people show a trend of increased stemness scores, although this does not necessarily indicate improved functionality.^205^^,^^206^ During aging, the HSC population expands primarily due to the clonal proliferation of dysfunctional HSCs that can still proliferate but exhibit impaired differentiation capacity. Age-related epigenetic reprogramming of HSCs affects both cancer-related and developmental pathways, potentially predisposing individuals to the development of age-related acute myeloid leukemia.^206^ Quiescent or senescent HSCs, although nondividing, are more resistant to cell death, adding to the accumulation of dysfunctional cells within the HSC pool. This accumulation may reflect an adaptive selection mechanism that helps inhibit the uncontrolled expansion of dysfunctional HSCs, thus maintaining some balance. However, senescent HSCs are more dysfunctional; they cannot regenerate and may actually promote tissue dysfunction through proinflammatory signaling. Over time, the dominance of dysfunctional HSCs in the hematopoietic system contributes to abnormal alteration of the cell composition in the blood and increased risks for age-related diseases, such as acute myeloid leukemia. Therefore, rather than being an adaptive selection specifically for improved self-renewal, the persistence of increased “stemness” during aging likely represents a pathological feature. Identifying age-related changes in cell identity and stemness could thus serve as novel biomarkers of immune aging.

However, several concerns remain regarding the current practical use of these biomarkers. Additionally, the identification and validation of novel biomarkers will require extensive research and longitudinal studies to confirm their reliability and clinical relevance.

To expand the current knowledge about immunological biomarkers of aging, future longitudinal studies should focus on integrating multiomics data, including genomics, proteomics, and metabolomics. Analyzing those comprehensive data over time could capture the dynamic changes within the aging immune system and will also give insight on how immune cell identity is altered with age. Longitudinal studies with larger cohorts are essential for validating the identified biomarkers and assessing trajectories and the predictive value for age-related health outcomes. Furthermore, exploring the impact of lifestyle interventions, such as diet and exercise, on immune aging could provide valuable insights into strategies for promoting healthy aging. By addressing these challenges and broadening our understanding of immunological biomarkers, we can develop more effective interventions to mitigate the effects of aging on the immune system and improve the overall healthspan of the elderly population.

## Data Availability

No new data were generated or analysed in support of this research.
